# Self-Assemblage and Quorum in the Earthworm *Eisenia fetida* (Oligochaete, Lumbricidae)

**DOI:** 10.1371/journal.pone.0032564

**Published:** 2012-03-01

**Authors:** Lara Zirbes, Yves Brostaux, Mark Mescher, Maxime Jason, Eric Haubruge, Jean-Louis Deneubourg

**Affiliations:** 1 Functional and Evolutionary Entomology, Gembloux Agro-Bio Tech, University of Liege, Gembloux, Belgium; 2 Applied Statistics, Computer Science and Mathematics, Gembloux Agro-Bio Tech, University of Liege, Gembloux, Belgium; 3 Department of Entomology, Center for Chemical Ecology, Pennsylvania State University, State College, Pennsylvania, United States of America; 4 Unit of Social Ecology, Free University of Brussels, Brussels, Belgium; University of Arizona, United States of America

## Abstract

Despite their ubiquity and ecological significance in temperate ecosystems, the behavioural ecology of earthworms is not well described. This study examines the mechanisms that govern aggregation behaviour specially the tendency of individuals to leave or join groups in the compost earthworm *Eisenia fetida*, a species with considerable economic importance, especially in waste management applications. Through behavioural assays combined with mathematical modelling, we provide the first evidence of self-assembled social structures in earthworms and describe key mechanisms involved in cluster formation. We found that the probability of an individual joining a group increased with group size, while the probability of leaving decreased. Moreover, attraction to groups located at a distance was observed, suggesting a role for volatile cues in cluster formation. The size of earthworm clusters appears to be a key factor determining the stability of the group. These findings enhance our understanding of intra-specific interactions in earthworms and have potential implications for extraction and collection of earthworms in vermicomposting processes.

## Introduction

The tendency of individual organisms to aggregate or disaggregate in space is a key aspect of social organization with far-reaching implications for population dynamics, ecology, and evolution [1]. The formation of more or less stable groups of individuals is observed in diverse organisms, ranging from microorganisms to vertebrates [2]–[5]. Such groups may arise when individuals converge at a particular location via independent responses to environmental factors [6] such as the spatial distribution of food resources, predation pressure, habitat quality, light and temperature gradients, or other abiotic and biotic variables [7]. Alternatively, group formation and cohesion may be driven mutual attraction or other social interactions among individuals [3], [4], [6], [8], [9]. For example, a self-assemblage is defined, most often in the context of social insect societies [10], [11], as a physical structure comprising individuals that have linked themselves to one another [10]. The stability and persistence of aggregations depends on the frequency with which new individuals join the group and existing members leave, and in the case of groups maintained by social cohesion, either or both of these processes may be influenced by group size (e.g. [8], [12], [13]). In some cases a quorum or a threshold emerges, and may be defined as a critical group size at which the collective dynamics of the group (e.g., the propensity of individuals to join or leave) change sharply rather than varying in proportion to the stimulus [14].

The specific mechanisms involved in group formation and maintenance vary considerably across organisms and can have significant implications for the structure and ecological functions of social groups [15]. However, these mechanisms are not always well understood, particularly in non-insect invertebrates. In *C. elegans*, for example, genes implicated in aggregation behaviour have been identified [16], but the detailed mechanisms of group formation remain unresolved. Very little work has addressed the social behaviours of earthworms, although intra-specific interactions, such as mating behaviour have been shown [17], [18], and a recent study reported coordinated movement among individuals *Eisenia fetida* (Savigny, 1826) [19].

**Figure 1:**
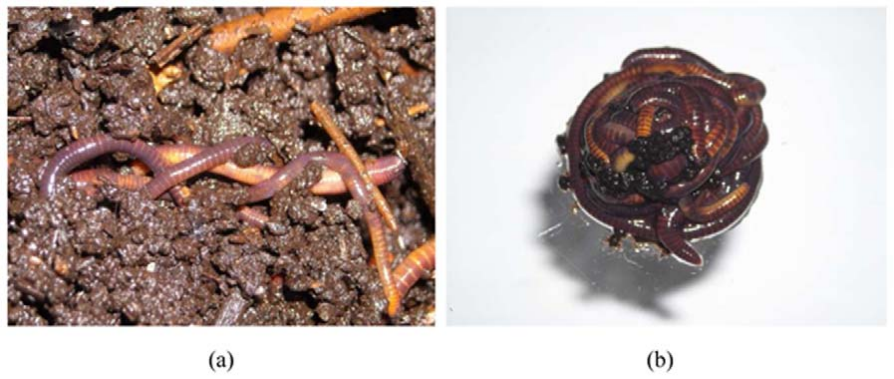
Earthworms clusters. (a) Earthworm group in a rearing box; (b) self-assembled earthworm cluster formed out of the soil.

The current study builds on that previous work by exploring the tendency of *E. fetida* individuals to join or leave groups and the implications for group maintenance through a combination of behavioural assays and mathematical modelling *E. fetida* belongs to the epigeic earthworms group [20], which live on or near the soil surface, typically in the litter layers of forest soils or organic rich materials (such as compost), and do not burrow [21], [22]. Epigeics feed on litter and/or the attached micro-flora and ingest little mineral soils [23]. A number of studies have documented the distribution of earthworm communities and the influence of soil physical and chemical factors on these distributions [21], [24]. In general, annelids exhibit patchy spatial distributions [25], and this is particularly the case for lumbricids, including *E. fetida* [18], [26], [27]. Aggregation is one factor that could contribute to a relatively uneven spatial distribution; moreover, many qualitative observations regarding annelids behaviour strongly suggest the possibility of intra-specific interactions and self-assembly [28], [29]. Our observation of apparently self-assembled clusters of *E. fetida* clusters in soil and rearing substrates in the laboratory (Figures 1a and 1b) motivated the research described here, which investigates the processes by which such aggregations are formed and maintained.

## Methods

### *Eisenia fetida* rearing

Earthworms (*Eisenia fetida*) provided by Ouroboros s.a. (Belgium) were reared in PVC boxes (42 cm long×30 cm wide×10 cm high) filled with universal compost DCM ® (De Ceuster Meststoffen s.a.,Grobbendonk, Belgium). The compost was changed every two months and cocoons and hatchling earthworms were placed in new boxes with fresh compost. Boxes were maintained at 23±1°C. Only mature earthworms (with a clitellum) were used for experiments.

### Experimental procedure

The ambient temperature for all experiments was 20±1°C and relative humidity was 62.5%. Experiments were conducted under red light in order not to disturb the earthworms [23].

#### Assays on group joining

**Figure 2:**
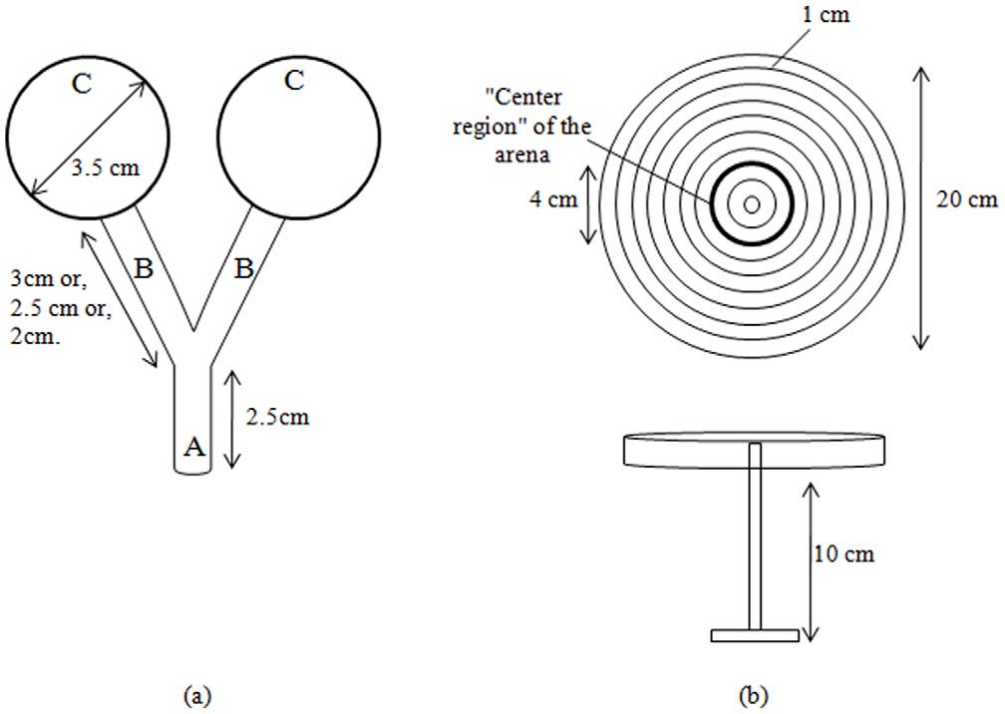
Y binary choice and arena set-ups. (a) Y device. A = 2.5 cm long neutral area where a single earthworm is placed at the beginning of the experiments, B = two lateral branches (2, 2.5 or 3 cm long), C = Circle chambers (3.5 cm diam.) where 15, or 0, earthworms were placed during the experiments; (b) Lateral and frontal view of the circular glass arena (20 cm diam.) with concentric circles (drawn on a transparent sheet affixed below) and indicating the central region (a circle of 4 cm diam.) the glass arena was elevated by a pedestal (10 cm).

To determine whether chemical compounds emitted by *E. fetida* attract conspecifics, a Y-tube choice assay was used. The set-up consisted of a semi-transparent Y-shaped Teflon device (Figure 2a), with 2 identical circular target chambers (diameter: 3.5 cm) connected via branch passages (diameter: 0.5 cm, 2, 2.5, and 3 cm lengths were used) to a 2.5 cm long passage into which earthworms were initially released. Mesh tissue placed at the junction between each target chamber and the branch passage prevented earthworms in the target chambers from escaping. For choice assays, 15 adult *E. fetida* were randomly selected from the rearing box, rinsed with tap water, and placed randomly in one of the circle chambers. An individual earthworm was then placed at the starting point of the set-up and its movement was documented using a numerical camera (JVC®, Everio GZ-MG333) over a 45-min period. The earthworm was considered to have made a choice when it touched the mesh tissue at the end of one of the branch passages. The target chamber selected, and the times taken to make a choice were recorded. We conducted 30 repetitions for each of the three branch lengths (2, 2.5 and 3 cm). As a control, we also conducted 30 repetitions with empty target chambers. Earthworms were removed and the set-up was washed with norvanol between trials.

A second behavioural assay employed a circular glass arena (20 cm in diameter), raised 10 cm by a pedestral to prevent earthworms escaping (Figure 2b). A transparent sheet marked with concentric circles at 1 cm intervals was affixed to the bottom of the arena. Adult *E. fetida* (0, 10 or 15 earthworms depending on the experiment) were taken from the rearing box, rinsed with tap water, and placed in the “centre region” of the arena (defined by the circle having a 2 cm radius from the centre of the arena). An individual earthworm was then placed midway between this centre region and the edge of the arena, and its movement was video recorded (as above) over a 45-minute period. We then recorded whether the earthworm either into the central region or to the edge of the arena (at which point the trail was deemed complete), as well as the time taken to initiate movement and the time to completion of the trial. We conducted 46 repetitions for each cluster-size (i.e., number of earthworms clustered in the central region: 0, 10 and 15). (As discussed below, groups of 10–15 earthworms are quite stable, and on only two occasions was a single worm observed to leave the central cluster during these assays. In these cases the departing worm was removed and replaced with a new one). The set-up was washed with norvanol between trials.

#### Assays on group leaving

Using the circular arena described above, adult *E. fetida* (1, 2, 5 or 10 earthworms depending on the experiment) were taken from the rearing box, rinsed with tap water, and placed within the centre region of the arena. Their movements were captured on video (as above) over 90 minutes. We then recorded the number of earthworms that left the central region and the time taken to leave. The experiment was repeated 30 times for each initial group size. The set-up was washed with norvanol between trials.

### Data analysis

For the Y-tube choice assay, a chi-square test for independence (Minitab® v15.0 State College, Pennsylvania USA - df = 2, α = 5%) was used to study the influence of branch lengths on earthworm choices, and a chi-square goodness-of-fit test (Minitab® v15.0, df = 1, α = 5%) was used to determine earthworm attraction by conspecifics. The influence of branch length and earthworm choices on earthworm times to choose was studied with a general linear model test with 2 factors (Minitab® v15.0, α = 5%).

For group-joining assays in the circular arena, a chi-square test for independence (Minitab® v15.0, N = 46, df = 2, α = 5%) was used to compare the number of earthworms reaching the central region when different numbers of earthworms were presented there. A general linear model with 1 factor (Minitab® v15.0, α = 5%) was used to compare times to initial movement and times to trial completion (when earthworm reached the central region or the edge of the arena) when different numbers of earthworms were presented.

For group-leaving assays in the circular arena, a one-way ANOVA test (Minitab® v15.0, α = 5%) was used to compare times until the first earthworm left the central region (N = 30). A difference test of the empiric survival curves estimated on the same data was also conducted to compare the kinetic of this assay (R 2.10.0, R Development Core Team, α = 5%). This test was also realised to study the departure of the second earthworm from each group. General linear model tests were used to compare leaving times of the second earthworm according to the initial group size and, to compare times departure of the second earthworm in a group of two when the second departing earthworm was partially dragged away from the group via contact with the first (a relatively common occurrence) and when there was no such interaction between the first two departing earthworms.

## Results

### Assays on group joining

Table 1 shows results for dual choice assays using the Y set-up. Distances (branch length) did not influence earthworm choice (Independence chi-square test, χ^2^_2_ = 1.92, p = 0.382), or earthworm time to choose (General linear model with 2 factors, F_2,62_ = 0.08, p = 0.92). We therefore grouped the 90 replicates. In general, we observed three typical behaviour patterns: In some cases, earthworms immediately moved from the start point and rapidly made a choice (i.e., moved down one of the branches and contacted the mesh tissue blocking passage to the target chamber); in other cases, earthworms move more slowly and explored the starting passage and one or both of the branches before making a final choice; finally, some earthworms made no choice, either remaining immobilised at the starting point or exploring the starting passage and branches without touching the mesh tissue. Groups of earthworms elicited strong attraction in this assay (>70% of earthworms chose the target chamber containing earthworms; Chi-square goodness-of-fit test: N = 68, χ^2^_1_ = 11.53, p = 0.001). An additional 30 trials conducted with no earthworm aggregate present revealed no evidence of bias in the Y set-up, and half of the tested earthworms made no choice under this condition (right = 7, left = 8, no choice = 15). In addition to eliciting a preference for the occupied target chamber, the presence of earthworm aggregates significantly increased the time taken to make a choice (12.9±1.1 min vs. 6.7±0.5 min when earthworms were absent; general linear model with 2 factors, F_1,62_ = 31.51, p = 0.005).

**Table 1:** Earthworm choices and choice times for behavioural assays in Y set-up.

	Experiment number				Average time (min) + SD			
	*3 cm*	*2.5 cm*	*2 cm*	*Grouped*	*3 cm*	*2.5 cm*	*2 cm*	*Grouped*
No choice	7	10	5	22	45	45	45	45
Group way	14	16	18	48	11.68±12.7	13.18±11.9	13.28±13.2	12.8±12.4
Other way	9	4	7	20	7.5±7.3	6.75±9.2	6.64±3.77	7.1±6.4
Total	30	30	30	90				

Attraction to aggregates was also observed in the circular arena. In these experiments, single earthworms generally began by exploring their immediate vicinity through movement of only the anterior part of the body (the head), and then initiated movement toward or away from the central region. Table 2 shows the total number of isolated earthworms that reached the central region or the edge of the arena for each aggregate size and the average time taken to complete the trial. Five earthworms did not reach either the edge or the central region within 45 minutes (in each of these cases, the earthworms remained immobile throughout the trial). Significantly more earthworms reached the central region of the set-up when earthworms were present there (Independence chi-square test, χ^2^_2_ = 13.095, p = 0.001). And the number of earthworm reaching the central region was linearly dependent of cluster size (y = 19.1×−2.73; r^2^ = 0.99). However, times to first movement did not vary significantly with aggregate size (Table 2; General linear model with 1 factor, F_2,130_ = 2.15, p = 0.12). Nor did time to reach the central region (General linear model with 1 factor, F_2,43_ = 0.16 p = 0.85).

**Table 2:** Results of joining assays in the circular arena: total number of isolated earthworms reaching the central region or the edge for each cluster size; and average times to the first movement and to arrival at the central region.

	Experiment number				Average time (min) ± SD	
	No choice	Centre of arena	Edge of arena	Total	First movement	Center of arena
No earthworm	0	7	39	46	3.5±2.8	7.6±5.0
10 earthworms	1	17	28	46	2.5±2.4	7.2±5.5
15 earthworms	4	22	20	46	3.8±3.6	8.5±7.3

### Assays on group leaving

**Figure 3:**
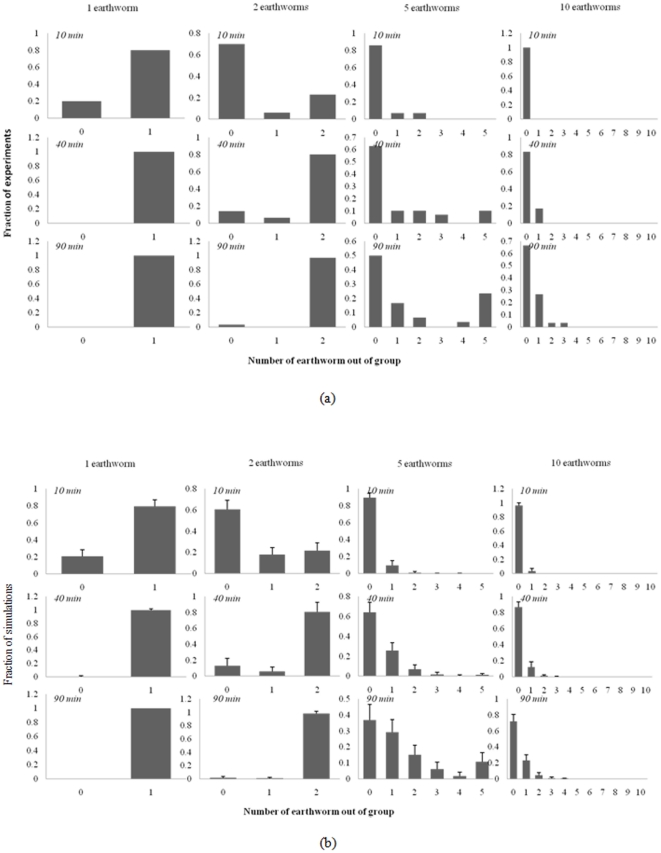
Departures of earthworms. Frequency distributions of observed (a) and expected (b) numbers of individuals leaving clusters of 1, 2, 5 or 10 earthworms over 10, 40 and 90 min.

**Figure 4:**
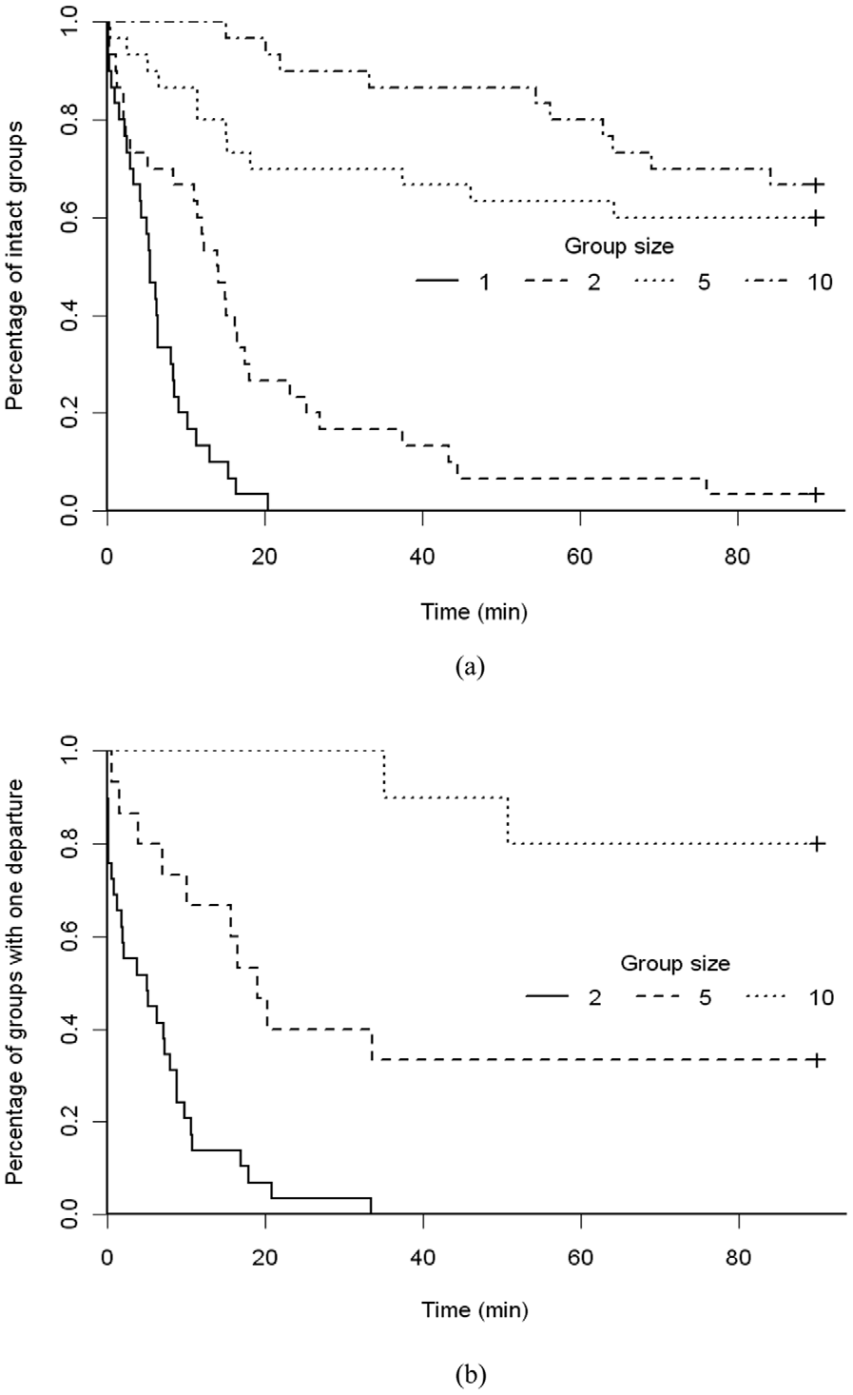
Survival curves for earthworm departures. Results of survival curve analysis for the departure of the first (a) and second (b) earthworms from groups of 1, 2, 5 or 10 earthworms; t_0_ for the second earthworm is the departure time of the first.

**Figure 5:**
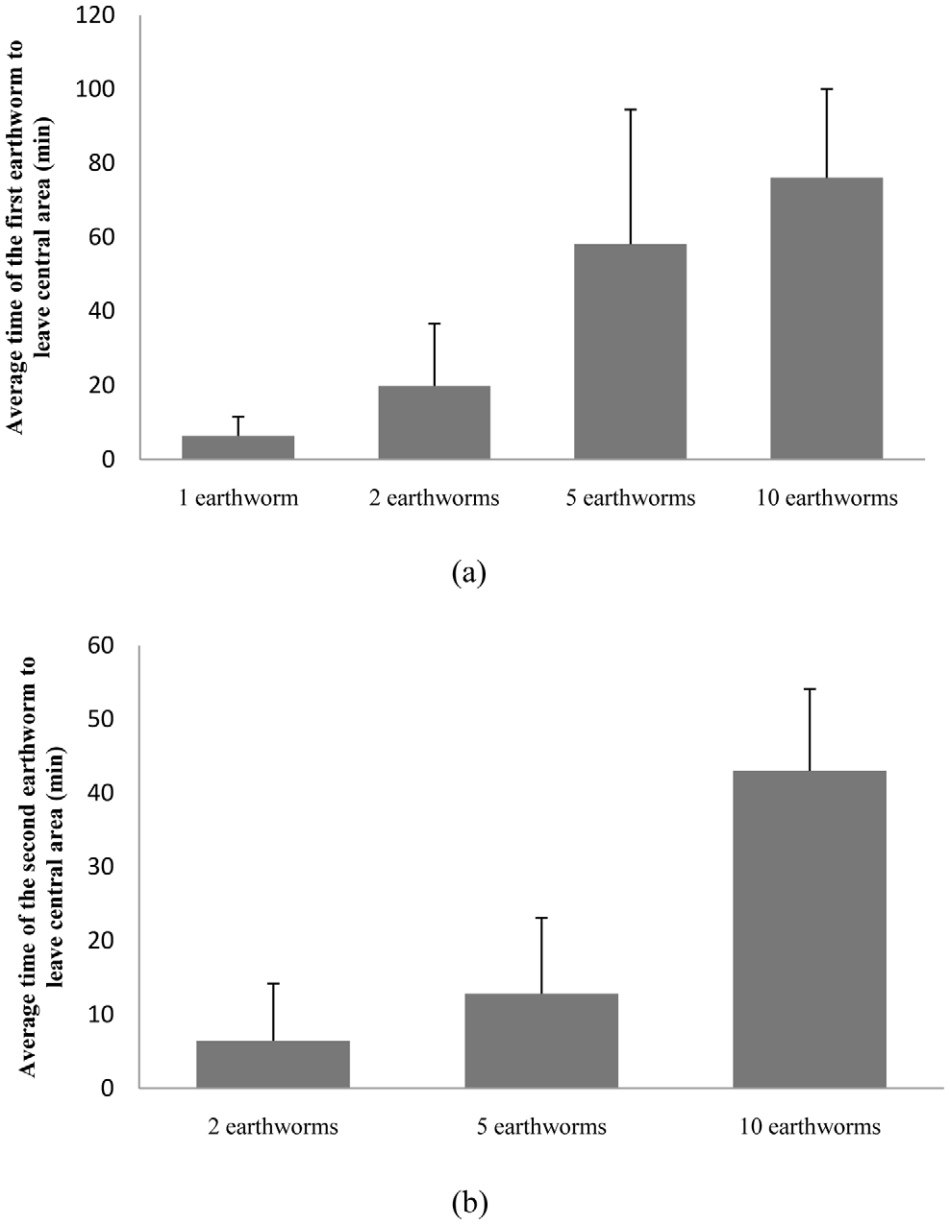
Average time for earthworm departures. Average time until the first (a) and second (b) earthworms leave the central region of the circular arena for group of 1, 2, 5 or 10 earthworms; t_0_ for the second earthworm is the departure time of the first.

Group-leaving assays in the circular arena revealed that the likelihood of group member departing changed over time and was significantly influenced by aggregate size. As shown in Figure 3a, the probability of leaving a group decreased as the number of earthworms present increased. Survival curve analysis (Figure 4a) likewise showed that earthworms were less likely to leave the central area when the size of the group increased (χ^2^_3_ = 107, p<0.001). The time taken for the first earthworm to leave the central region also increased aggregate size (One way ANOVA, F_3,116_ = 54.47, p<0.001). On average, a single earthworm left the central region in less the 1/12^th^ the time it took the first earthworm to leave a group of 10 individuals (Figure 5a).

#### Equations to describe earthworms' behaviour

**Figure 6:**
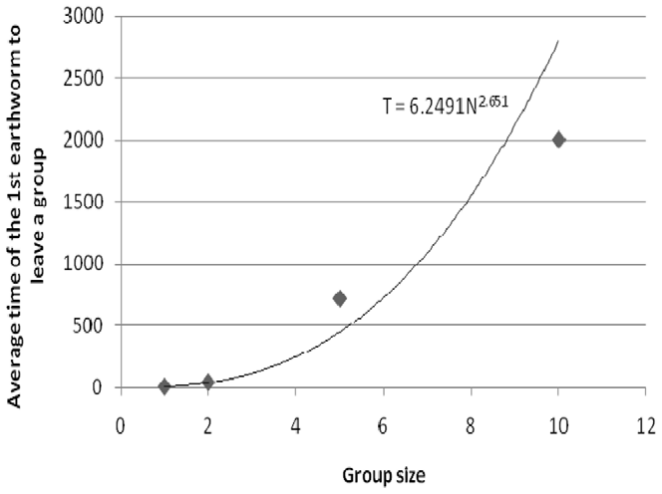
Time equation of the first earthworm's departure. Average time to departure is a function of the cluster size and its adjustment (black line).

In this context, the survival curve of the intact groups (without any departure) (Figure 4a) was approximated by the exponential equation:
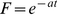
(1)where *F* is the fraction of groups without any departure at time *t*, and *a* is the inverse mean time of the first departure and corresponds to the probability of leaving. Using this approximation, the individual average time of the first earthworm to leave a group (T) can be calculated for each earthworm population (N) using the equation:
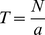
(2)where the time of the first earthworm to leave a group increases with the number of conspecifics (N) in the group. Based on figure 6, the equation to express the departure time of the first earthworm in function of the earthworm population size was determined to be:
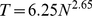
(3)Similarly to that of the first earthworm, the probability of a second earthworm leaving a group significantly decreased with increasing aggregate size (Figure 4b; χ^2^_2_ = 35.8, p<0.001), while the time taken to leave increased (Figure 5b; General linear model, F_2,38_ = 18.0, p<0.001). As the time to the second earthworm departure is measured from the first, t_0_ for the second earthworm is the departure time of the first earthworm to leave the group. Sometimes an earthworm left the central region in contact with the previous earthworm (i.e., was partially dragged by it). It is straightforward to quantify this effect in groups of 2 earthworms. Departure of the second earthworm was significantly more rapid when contact between earthworms was observed, 0.39 min±0.68 min (mean ± SD) vs. 8.71 min±8.08 min (mean ± SD) when contact was not observed (General linear model, F_1,27_ = 20.45, p<0.001). Due to contact between individuals, the survival curves for the second earthworm leaving the centre region can be estimated as a double exponential:
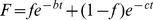
(4)where *f* ( = 0.24) is the fraction of departures with contact, (1-*f*) is the fraction of contactless departures, *b* is the constant of departure by contact (*b* = 7.32 min^−1^), and *c* ( = 0.11 min^−1^) is the constant of contactless departure. With assuming constant probabilities, the inverse of *b* and *c* correspond to mean delays between the two earthworm departures (with and without contact, respectively). Thus, *b* ^−1^ and *c* ^−1^ correspond to the mean duration of the second earthworm departure (with contact 0.14 vs. 0.39 min; without contact 9.1 vs. 8.71 min).

#### Model to describe earthworms' behaviour

The objectives of the following model were to validate the agreement between our observations at the individual and collective levels and to highlight some characteristics of the collective dynamics such as the emergence of a quorum or threshold group size.

Our experimental results and analyses indicate that the individual average time of the first earthworm to leave a group increases with the number of conspecifics (N) in the group (see equation 2). The survival curve of intact groups being approximated by exponential, the individual probability of leaving was therefore the inverse of this average time (see equation 1). We assumed that the probability of leaving was the same for each individual and equal to Q(N), where *N* is the number of individuals in the aggregate:
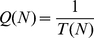
(5)Moreover, for the followers (the next to leave), we neglected the facilitation effect due to the departure of a previous earthworm (see equation 4).

To summarize the model, we assumed a continuous time Markovian jump process, that is, the probability, per time unit, of the response occuring (i.e., leaving the aggregate) is constant as long as the stimulus (i.e., the size of the group) remains the same, but jumps to a new value when the stimulus changes (i.e., when an earthworm leaves the aggregate).

To test the relevance of the parsimonious model and understand the main effects arising from the dynamic fluctuations, Monte Carlo simulations were used, in which the random aspect of the process is automatically incorporated. Simulations were based on the previously estimated probability of leaving a group Q(N), being the inverse of mean leaving time. We assumed that each individual obeyed this function. The steps of the model can be summarized as follows: (1) initial conditions: the number of individuals within the group (N) was determined at N_0_; (2) decision process: At each time step (t), the position of each individual (remaining within the group or outside it) was noted. Then the probability of moving out of the cluster is then given by Q(N) for each individual in the group. The departure of an individual at time t depends on the comparison between the calculated value of Q and a random number sampled from a uniform distribution between 0 and 1. If this value is less than or equal to Q, the individual leaves the cluster. If not, it stays within it. The probability Q(N) of leaving the group was updated at each simulation step in relation to the number of individuals remaining. In the model an earthworm will never re-join the cluster (no entry). Iteration of this process allowed us to simulate the survival of cluster over time, and the process was repeated for 90000 steps (i.e., 180 minutes, where each time step = 0.01 min). Monte Carlo simulations were run 6000 times (200× groups of 30 simulations). The distributions of the numbers of individuals present within the cluster were calculated in relation to time and are compared to the experimental results.

There was good agreement between the theoretical and experimental results for group of 1, 2 and 10 earthworms and weaker agreement for groups of 5 (Figures 3a and 3b). Isolated individual quickly leaves the initial zone and during the experimental time; for group of 10 worms, most of the earthworms remain within the cluster. For 2 and 5 earthworms, the distribution of the number of simulations and experiments as a function of the number of earthworms having left is bimodal: roughly, the simulations can be divided into a class with a small number of earthworms having left the cluster and a class with all individuals having left. These dynamics result from the dependence of the probability of leaving the cluster Q(N) on the cluster size (N) and imply the presence of a threshold group size: depending of the initial population, the system exhibits qualitatively different responses.

**Figure 7:**
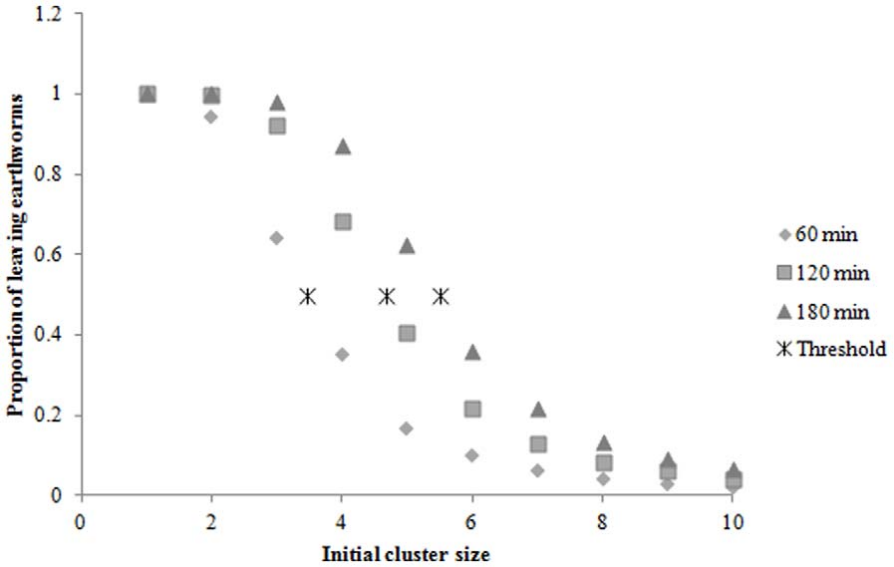
Mean proportion of earthworms leaving a cluster over 60, 120 and 180 min. as a function of the initial size of the cluster. * denotes threshold for 60, 120 and 180 min.

The existence of such a threshold, or quorum, is also indicated by the mean proportion of earthworms having left before a given time, t. This mean proportion (F calculated from 1000 pooled simulations) decreases following a sigmoid curve when the initial size of cluster (N_0_) increases and changes sharply when the initial number of earthworms crosses a threshold (S) (Figure 7). At any time t, this mean proportion is well fitted by a Hill-type function [14] (r^2^>0.99, 3.2<k<5.65, 1.5<S<6.7 for 10, 20,…,180 min):
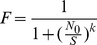
(6)Following Sumpter and Pratt [14], *F* is a quorum response at the level of the cluster. The threshold value *S* can also be defined as the value of N_0_ that gives a mean proportion of individuals having left the cluster = 0.5; *k* determines the steepness of the function *F*. S and k are function of time t: larger values of t yield larger values of S and k. Figure 7 shows that the threshold increased gradually with time. For example, the threshold was about 3.5 earthworms over 60 min and about 5.5 earthworms over 180 min. This threshold emerged at the collective level from the dynamics of departure. Indeed, the individual probability of leaving Q(N) did not exhibit any threshold behaviour.

**Figure 8:**
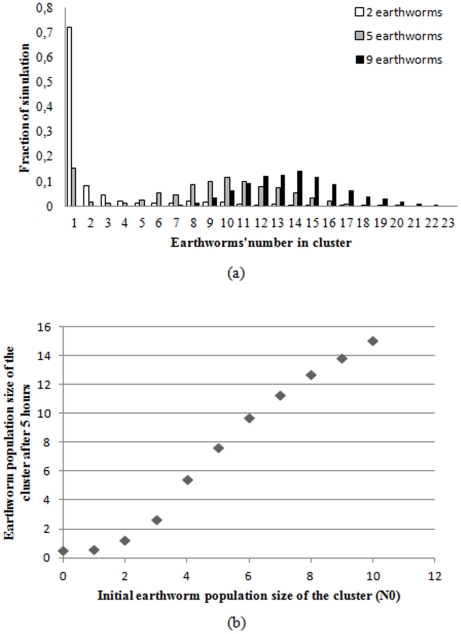
Thresholds of the aggregate. (a) Distribution of cluster size at 5 hours for three different initial populations: 2 earthworms, 5 earthworms, and 9 earthworms, with the probability of joining μ = 0.02 min^−1^ and that of leaving Q(N) = N^−2.6^/6.25 min^−1^. (b) Mean cluster size at 5 hours as a function of the initial size with the probability of joining (μ) = 0.02 min^−1^ and that of leaving Q(N) = N^−2.6^/6.25 min^−1^.

Furthermore, when the model is modified to account for joining as well as leaving and therefore for the possibility of increases in group size it is easy to show that the system exhibits an initial critical size (or threshold) of the aggregate (Figures 8a and b). In this version of the model, we assumed that the group is surrounded by a constant population of earthworms, from which an earthworm joins the cluster with a constant probability per time unit (μ). This parameter combines the movement speed of the earthworms and their surrounding density. Concurrently, each earthworm in the aggregate at a given time step may leave with the probability Q(N). These simulations started with an initial number of earthworms (N_0_) and the time of the simulation was 5 h. The sigmoidal shape of the mean population within the clusters as a function of the initial population clearly confirmed the existence of the threshold. This threshold effect was apparent in the distribution of the simulations outcomes as a function of initial group size (Figure 8a). The threshold value is estimated as the value of N_0_ for which the entrance μ is equal to the departure N_0_Q(N_0_) (for the simulated values N_0_≈4); lower the probability of joining (μ), the greater the threshold. For small starting aggregates (2 individuals) below the threshold, the peak occurs at N = 0, indicating that most of the clusters collapsed. For larger starting aggregates peaks emerged respectively at N = 0 and some larger value of N. For example, for groups of 5, the second peak occurred at N = 10 (Figure 8a). In this case, where the initial value (N_0_ = 5) was close to the threshold, some aggregates increase in size and others collapse and very few remain at the initial size. For values of N_0_ (e.g. 9 individuals) well above the threshold, almost all aggregates increased in size and the distribution exhibits a single peak (Figure 8a).

The threshold dynamic can also be seen if the mean size of the cluster reached after time unit t is presented as a function of the initial cluster size. In this case, mean group size remains close to zero for small initial cluster size (N_0_) and increases abruptly as N_0_ becomes larger (Figure 8b).

## Discussion

Our results clearly demonstrate the existence of attractive cues that promote the formation and maintenance of multi-individual groups in *E. fetida*. To our knowledge, it is the first demonstration of complex social structure in earthworms. As attraction occurs at a distance, it is likely to be mediated by olfactory reception of volatile cues, possibly an as yet unidentified aggregation pheromone. Once formed, earthworm assemblages may be maintained by the same or other chemical cues and/or by tactile cues. Moreover, both attraction to and retention within groups are stronger for larger aggregates, suggesting a quantitative response to the cues involved.

While volatile signals and cues are known to mediate a wide array of interactions within and between species [30], most previous work on volatile infochemicals has focused on aboveground interactions [31]. However, volatile chemicals are also present underground and can be disseminated over some distance via air-filled pores in the soil matrix [30]. Some recent work has begun to document the role of volatile cues in below-ground ecological interactions, for example among plant roots, nematodes and arthropods [32]. But very little work has addressed the use of volatile cues by earthworms, though these organisms are known to possess chemoreceptors, located principally on the prostonium or on the buccal epithelium [21]. Pheromonal signalling was previously implicated in the induction of thermo-tolerance in *Tubifex tubifex* and *Enchytraeus albidus*, two aquatic annelids [33]. And, we recently reported that earthworms are able to use olfactory cues to actively search for food microbial sources [34].

In the current study, the presence of earthworm assemblages in our Y-choice assay elicited attraction, but also increased the time taken by individual earthworms to make a choice relative to that observed when both target chambers were empty (12.9±1.1 min vs. 6.7±0.5 min). Klinotaxis, which has been observed in many animal phyla [35], is a potential explanation for the increased time to chose when presented with an attractive stimuli—for example, if worms initially selecting the control branch of the Y-tube experience a decreasing concentration of the signal and subsequently turn back. Alternatively, earthworms might initiate active complex searching behaviours in response to detection of the group-derived cue perhaps exhibiting stop phases and side-to-side movement, as seen in some insects [36] and nematodes [37]. Visual inspection of the videos taken during our trials provides some evidence for both these factors, but was not sufficient to draw rigorous conclusions about their relative importance.

Another key finding of our study is that the stability of aggregates increases with size. Similar dynamics have previously been reported for insects (e.g., aggregations of cockroaches under shelters) [12], [38], [39]. Jeanson et al. [40] studied aggregation site selection by the ant *Messor barbarous* and found that the probability of ants leaving a selected site decreases with the number of workers at the site. In another ant, *Lasius niger* greater numbers of ants inside a cluster decreased the probability of individual ants leaving [41] .

Modelling allows us to explore such dynamics by the testing the effect of various rules of interaction based on minimal hypotheses and determining whether the resulting simulations yield outcomes similar to those observed through experimentation [6]. Good agreement between our theoretical (model) and experimental results confirms that the probability of leaving a group decreases with the number of earthworms in a cluster; however, we observed some differences between our theoretical and observed distributions of earthworms leaving over time. Most significantly, from group of 5 earthworms, we observed no departure after 90 min in 50% of experiments, whereas our simulations predicted 35%±10% (SD); and in 25% of the experiments all 5 earthworms departed over this time period, whereas the simulation predict 11%±5% (SD). These discrepancies can likely be explained by the role of contact between earthworms in accelerating departure (i.e., contact with a departing worm increases the probability of departing), which we quantified for groups of 2 and 5 earthworms. This explanation is enhanced by a previous study on *E. fetida* which demonstrated that contact among individuals plays an important role in coordinating the direction of movement [19]. When both joining and leaving were incorporated in the model, simulations revealed a critical threshold level for the initial group size below which aggregations were apt to collapse and above which they survive and increase size The value of the threshold depends on the probability of joining (μ). Further elaboration of this model in conjunction with empirical studies may allow us to understand how such factors influence the spatial organization and dynamics of natural earthworm populations.

The significance of aggregation for earthworms is currently not well understood. In general group formation can provide advantages by facilitating information transfer between individuals [42]; increasing success in resource (e.g., food) acquisition [43]; enhancing resistance to, or regulation of, environmental conditions (e.g., temperature and humidity) [44]–[46]; or improving defence against predators [47], [48]. With respect to defence, enhancement of chemical defences could be one advantage of aggregation in earthworms [49]. For *E. fetida*, these defences involve specialized cells that float in the coelomic fluid and secrete humoral effector proteins. Coelomic fluid has been shown to exhibit cytolytic and anti-bacterial activities that are believed to play a role in defence against soil pathogens [50]. Moreover, *E. fetida* secrete coelomic fluid in response to attack by the flatworm (*Bipalium adventitium)*, eliciting an aversive response in the flatworms and increasing the survival rate of earthworms [51]. Shared or coordinated defence might be particularly beneficial when earthworms are under high predation pressure, as is often the case for *E. fetida* [22] Increased resistance to adverse environmental conditions (e.g. flooded or dry soil) could be another advantage of aggregation in earthworms [21]. By forming an aggregate, the earthworms reduce their collective surface-to-volume ratio and may reduce their vulnerability to different stresses. Both humidity and temperature have previously been identified as important factors contributing to the composition and the structure of earthworm communities [52], [53]. In mites, which particularly vulnerable to dehydration, cluster formation helps to reduce water loss (Gloss, 1998). Aggregation could be an initial step in coordinated migration. As noted above, coordinated movement in *E. fetida* has been recently reported [19], and Doeksen [54] observed that *E. fetida* living greenhouse soil migrated up the sides of buildings in large numbers during damp, foggy weather. Mass migration has also been observed in earthworms in response to flooded soil conditions [55], and it has been speculated that this response promotes gene exchange within the population via active dispersal [56].

In conclusion, this study provides the first documentation of complex social organization in earthworms and indicates that olfactory cues play a key role in promoting aggregation. Moreover, group size appears to be a key factor contributing to the stability and persistence of groups over time. While there are many potential benefits of coordinated group behaviour, including collective defence, resource acquisition, and regulation of micro-environmental conditions, the ecological significance of aggregation in earthworms remains to be elucidated through further experimentation. Likewise, the specific cue or cues responsible for intra-specific attraction and the mechanisms by which earthworms perceive and respond to these cues remain to be discovered. Future work on these topics will significantly enhance our understanding of the ecology of earthworms, which are critical components of soil ecosystems in temperate regions. Furthermore, improved understanding of the mechanisms governing earthworm aggregation and the attractive cues involved could have considerable significance for the development of enhanced techniques for the extraction and sampling of earthworms in vermicomposting and other applications.
